# Supplementary material for: Esr1-dependent signaling and transcriptional maturation in the medial preoptic area of the hypothalamus shape the development of mating behavior during adolescence
Source: eLife. 2026 Jun 19;14:RP106347. doi: 10.7554/eLife.106347 (PMC13282117; doi:10.7554/eLife.106347)
Supplement: Peer review history

# Editor report: eLife Assessment

**DOI:** 10.7554/eLife.106347.3.sa0

**Authors:** Alexander Theodore Chesler

**Affiliations:** National Institutes of Health United States

**Keywords:** Convincing, Valuable

The authors test the hypothesis that gonadal steroid signaling influences the transcriptional development of specific neurons in the mPOA during adolescence, and that such adolescent development of the mPOA is necessary for mating behaviors. The **valuable** findings are supported by **convincing** evidence. This work contributes new insight into hormone-sensitive transcriptional profiles within genetically defined neuron clusters in the mPOA during adolescence and will be of interest to systems and molecular neuroscientists and those interested in development, sex differences, and/or hormonal regulation.

# Referee report: Reviewer #2 (Public review)

**DOI:** 10.7554/eLife.106347.3.sa1

Summary:

An abundant literature documents molecular changes in the rodent hypothalamus that occur during the transition from prepubertal to mature reproductive physiology. Equally well documented is the role of sex steroids and their receptors during this important period of reproductive development, as well as the importance of GABAergic and glutamatergic neurons. The medial preoptic area (MPOA) is known to play a central role in expression of sexually dimorphic reproductive function and previously reported sexually dimorphic patterns of gene expression are consistent with this role. The present manuscript extends this knowledge base and reports the results of a detailed evaluation of transcriptional dynamics in the MPOA during the adolescent transition to maturity with a particular focus on the role of the estrogen receptor gene (Esr1). Both single cell RNA sequencing (scRNseq) and multiplex in situ hybridization methods were employed and the results subjected to detailed computational analyses to demonstrate that the transcriptomic structure of MPOA neurons displays both sex and cell type specific expression profiles. In addition, both hormonal and genetic manipulations of Esr1 signaling during puberty altered the transcriptional profiles of MPOA neurons, and these changes aligned with maturation of hormone-dependent reproductive function. The authors provide this evidence to illustrate Esr1-dependent control of gene regulatory networks required for normal expression of reproductive behaviors expressed during the transition from adolescence to adulthood. The results presented in this manuscript are extensive and represent the most comprehensive evaluation of transcriptomic changes during reproductive maturation to date. The methods appear strong and the results provide a rich data set that will support a good deal of future analysis.

Strengths:

(1) The major strength of this manuscript is the extensive set of images and graphs that illustrate molecular changes that occur in MPOA neurons during adolescence, although additional spatial detail as to locations of the source neurons would be welcome in order to place the changes in the proper circuitry context.

(2) Targeting Esr1 deletion to MPOA GABA neurons is a good choice, given how these cells have been implicated in sexual differentiation of reproductive behavior previously, and the lack of comparable responses in glutamatergic neurons is convincing. The AAV-frtFlex-Cre virus created by the investigators is a most useful tool for such studies. Profiling distinct transcriptomic trajectories in GABA and glutamatergic neurons during reproductive maturation is impressive and leads to some of the best supported conclusions in this paper.

(3) Cellular and molecular resolution of the transcriptomics data appears excellent, however, because the source tissue for the scRNAseq analysis was obtained by bulk dissection of the MPOA anatomical resolution is limited. This problem is addressed to some extent by careful comparison of scRNAseq results with previously published spatial transcriptomics data. The HM-HCR-FISH analysis clearly documents spatially restricted changes in gene expression, but it is hard to discern where these changes occur based on the images presented or the descriptions included in the Results. The anatomical schematic included in Figure 4 suggests that investigators are not familiar with components of the MPOA (see Allen Mouse Brain Atlas).

Weaknesses:

(1) A major conceptual flaw is that the authors do not distinguish between genetically determined sex differences in patterns of gene expression and differences caused by the fact that MPOA neurons are exposed to different endocrine environments in adolescent males and females, which can cause different transcriptional trajectories independent of genetic sex. This issue does not render their results invalid, but their terminology should address the issue in the discussion and "limitations" section. At the very least the endocrine status of "intact females" should be included.

(2) A major technical flaw is that the MPOA is treated as a functionally distinct brain region (block dissections) with uniform distribution of cell types (FISH data are not illustrated or reported with sufficient spatial detail). Thus, an enormous amount of molecular data is provided that cannot be mapped to distinct neural circuits, thereby limiting the neurobiological impact. This is also a weakness of the FISH data, which is presented with only small regions illustrated without anatomical detail. In fact, some images are compared that appear to illustrate different MPOA structures, although it is impossible to be certain of this due to the lack of morphological landmarks. The analysis of how Esr1 orchestrates regulatory gene networks is impressive and interesting, but the fact that many of the observed transcriptional events occur in neural circuits that do not overlap confounds interpretation.

(3) The locations of the AAV injections should be characterized because deleting Esr1 in multiple distinct parts of the MPOA will likely confound interpretation. This is especially problematic given the limited number of mice used for parts of the RNAscope analysis.

(4) Although the focus of these experiments on adolescence is welcome, neither the Introduction nor the Discussion do a good job of placing these studies in the context of what is already known about brain maturation during puberty. It is true that this is very much a results-focused manuscript, but the scholarship can be improved. Simply stating that your results are consistent with previous reports places an undue burden on the reader to go figure out what is new.

(5) Throughout the manuscript, the authors utilize obscure abbreviations, which often makes reading their text overly cumbersome. This is certainly justified in certain instances where complex names of analytical methods are used repeatedly, but the authors are encouraged to try and simply their use of non-standard abbreviations.

Comments on revisions:

The authors have considered issues raised during the initial review. Although there do not appear to be significant changes to analyses, figures or conclusions, the authors have added important revisions listing limitations in study design and methodology that impact interpretation.

# Referee report: Reviewer #3 (Public review)

**DOI:** 10.7554/eLife.106347.3.sa2

The paper identifies effects of gonadal hormones within hormone-responsive GABAergic neurons in the MPOA. Although it is not surprising that hormones have effects on neurons that express hormone receptors, the current paper adds insights with higher cellular and spatial resolution than previous work and focuses on adolescence period. The paper also identifies a major role for Esr1-dependent mechanisms on behavior using an intersectional genetic strategy to ablate Esr1 in GABAergic or glutamatergic neurons in the MPOA.

The authors have thoughtfully addressed the reviews, in particular by focusing quantitative analyses on Vgat+Esr1+ clusters and adding important technical and conceptual considerations in the limitations section.

I have one remaining minor concern. I appreciate that the text now defines "transcriptional maturation". However, the term seems inappropriate when describing the "minimal transcriptional changes" in Vgat+hormone RLow clusters, which implies that they are transcriptionally immature. Do the authors mean to imply that transcriptional maturation is observed in Vgat+Esr1+ clusters but not Vgat+hormone RLow clusters? The authors also use the term "hormone-dependent transcriptional dynamics", which I think is more appropriate. For example, hormone-dependent transcriptional dynamics are observed in Vgat+Esr1+ clusters but not Vgat+hormone RLow clusters.

# Author comment: Author response

**DOI:** 10.7554/eLife.106347.3.sa3

**Authors:** Koichi Hashikawa, Yoshiko Hashikawa, Brandy Briones, Kentaro K Ishii, Yuejia Liu, Mark Rossi, Marcus L Basiri, Jane Y Chen, Omar Ahmad, Rishi Mukundan, Nathan Johnston, Rhiana Simon, James Soetedjo, Jason Siputro, Jenna McHenry, Richard D Palmiter, David Rubinow, Larry S Zweifel, Garret D Stuber

**Affiliations:** University of Washington Seattle United States; New York University New York United States; University of Washington Seattle United States; University of Washington Seattle United States; University of Washington Seattle United States; University of Washington Seattle United States; University of Washington Seattle United States; Howard Hughes Medical Institute, University of Washington Seattle United States; University of Washington Seattle United States; University of Washington Seattle United States; University of Washington Seattle United States; University of Washington Seattle United States; University of Washington Seattle United States; University of Washington Seattle United States; Duke University Chapel Hill United States; Howard Hughes Medical Institute, University of Washington Seattle United States; UNC Chapel Hill Chapel Hill, NC United States; University of Washington Seattle United States; University of Washington Seattle United States

The following is the authors’ response to the original reviews.

**Public review:**

**Reviewer #1 (Public review):**

Weaknesses:

Two minor comments

(1) Fig 4 (hormone treatment): In this experiment, testosterone is given to males, yet in Sup Fig 6 it is argued that Esr1 is more influential in driving transcriptional changes compared to AR. Does DHT treatment have the same outcome as testosterone? Or, does estrogen treatment in males have the same outcome as testosterone?

We agree that to distinguish AR and Esr1 activation by testosterone and converted estrogen respectively is a limitation in our study. We added discussion in the “limitation of the study” section.

Although HM-HCR experiments showed the bidirectional control of transcriptional progression during adolescence, it is unclear if the facilitation in male by testosterone supplement is via activation of *AR* or *Esr1* or both because testosterone will likely be converted to estrogen in the brain. Future studies using dihydrotestosterone (DHT) and estrogen to males may address this issue.

(2) Fig 3i: There appears to be an age-dependent transcriptional change in male Vgat HR-low cells. Can the authors comment on age-dependent (hormone-independent) transcriptional changes in males versus females.

We agree that it is important to clarify hormone dependent changes and age dependent changes. We added pair-wise DE results in Vgat HR low population in the main text. As consistent with trajectory analysis, the number of age-dependent genes were fewer than hormonally associated genes.

“Pair-wise DEG analysis consistently showed that larger number of *DEGs* between *P35* and *P23* in *Vgat*+*Esr1*+ (male: 146 genes; female: 162 genes) than *Vgat*+ hormone R^Low^ (male: 26 genes; female: 1 gene).”

**Reviewer #2 (Public review):**

Weaknesses:

(1) A major conceptual flaw is that the authors do not distinguish between genetically determined sex differences in patterns of gene expression and differences caused by the fact that MPOA neurons are exposed to different endocrine environments in adolescent males and females, which can cause different transcriptional trajectories independent of genetic sex. This issue does not render their results invalid, but their terminology should address the issue in the discussion and "limitations" section. At the very least the endocrine status of "intact females" should be included.

We agree that this was ideal if perinatal and pubertal dynamics are analyzed within the same study to distinguish these two processes. We added discussion in the “limitation section”.

“2. Although we have identified hormone/*Esr1* dependent transcriptional trajectories during adolescence, the relations and interplay with genetically determined perinatal event, which is earlier and robust, are unclear. Some sex differences during adolescence might be an extension of perinatally established sex differences while others might be unique adolescent changes.”

(2) A major technical flaw is that the MPOA is treated as a functionally distinct brain region (block dissections) with uniform distribution of cell types (FISH data are not illustrated or reported with sufficient spatial detail). Thus, an enormous amount of molecular data is provided that cannot be mapped to distinct neural circuits, thereby limiting the neurobiological impact. This is also a weakness of the FISH data, which is presented with only small regions illustrated without anatomical detail. In fact, some images are compared that appear to illustrate different MPOA structures, although it is impossible to be certain of this due to the lack of morphological landmarks. The analysis of how Esr1 orchestrates regulatory gene networks is impressive and interesting, but the fact that many of the observed transcriptional events occur in neural circuits that do not overlap confounds interpretation.

We agree that while MPOA is defined based on brain atlas consistently across samples, the boundary is somewhat less obvious compared to other nuclei (e.g. hippocampus, VHM etc). To minimize the contaminations from adjacent areas, we have restricted quantitative analysis to mostly *Vgat+ Esr1*+ population which are densely located within the MPOA but not in immediately adjacent areas, except posterior BNST which is readily distinguishable. We added clarification in the method as well as added technical limitation in the discussion below.

Method

“To disambiguate the MPOA and adjacent brain regions, quantitative analysis is restricted to *Vgat+ Esr1*+ neurons and is devoid of posterior BNST.”

Discussion

“3. While we have observed robust effect of *Esr1-KO* in scRNAseq experiment which was further validated with FISH experiment, it is possible that there are further heterogeneous *Vgat-Esr1* populations in the MPOA which might be differentially targeted in each virally injected sample. To mitigate this, 3-4 mice were pooled for each sample in scRNAseq experiment and in HCR-FISH experiment, in addition to confirming recombinase RNA expression within the MPOA, we included samples with robust *Esr1* deletion in the MPOA. Interestingly, due to the technical challenge, *Esr1* deletion tends to be more robust than weakly detected recombinase RNA expression (data not shown).”

(3) The locations of the AAV injections should be characterized because deleting Esr1 in multiple distinct parts of the MPOA will likely confound interpretation. This is especially problematic given the limited number of mice used for parts of the RNAscope analysis.

We agree that similar to #2, this is an important matter. For HCR experiment, we only included animal with recombinase RNA (Cre or Flp) expression within MPOA. Although the recombinase expression was sufficient enough to qualitatively determine the hit or miss, the detection was weak and it was challenging to determine the extent of viral spread. Thus, we also used successful Esr1 deletion as an additional inclusion criteria for AAV-Cre-YFP group. We have added inclusion criteria in the method and technical consideration in discussion.

Method

“For *HCR2*, AAV was injected unilaterally so that successful targeting of the MPOA with AAVCre-YFP (detection of recombinase RNA within the MPOA) and the deletion of *Esr1* were confirmed for inclusion of samples.”

Discussion

“3. While we have observed robust effect of *Esr1-KO* in scRNAseq experiment which was further validated with FISH experiment, it is possible that there are further heterogeneous *Vgat-Esr1* populations in the MPOA which might be differentially targeted in each virally injected sample. To mitigate this, 3-4 mice were pooled for each sample in scRNAseq experiment and in HCR-FISH experiment, in addition to confirming recombinase RNA expression within the MPOA, we included samples with robust *Esr1* deletion in the MPOA. Interestingly, due to the technical challenge, *Esr1* deletion tends to be more robust than weakly detected recombinase RNA expression (data not shown).”

(4) Although the focus of these experiments on adolescence is welcome, neither the Introduction nor the Discussion do a good job of placing these studies in the context of what is already known about brain maturation during puberty. It is true that this is very much a results focused manuscript, but the scholarship can be improved. Simply stating that your results are consistent with previous reports places an undue burden on the reader to go figure out what is new.

We agree that contextualizing our study in the scholarship will clarify the novelty and impacts that this study provides to the community. We have updated the introduction adding a review highlighting puberty associated genomic studies in the brain, which are all bulk (brain region level) as well as the very first puberty scRNAseq study in Human testis.

“Despite the well-established role of these hormones in shaping behavior, the molecular mechanisms underlying their influence on brain development during adolescence are still limited to brain-region level (bulk)[8]in humans and model organisms and adolescent transcriptional dynamics at single cell resolution in the brain remain poorly understood (but see a pioneering study in the human testis[9]).”

(5) Throughout the manuscript the authors utilize obscure abbreviations, which often makes reading their text overly cumbersome. This is certainly justified in certain instances where complex names of analytical methods are used repeatedly, but the authors are encouraged to try and simplify their use of non-standard abbreviations.

We agree that this is helpful for readers to have the reference of abbreviations in handy at single location. We added an “abbreviation” section as a reference for readers.

Medial preoptic area (MPOA)

Single-cell RNA sequencing (scRNAseq)

*Estrogen receptor 1 (Esr1)*

GABAergic neurons (Vgat+)

Glutamatergic neurons (Vglut2+)

Hybridized chain reaction fluorescent in situ hybridization (HCR-FISH)

Gonadectomized (GDX)

Partition-based graph abstraction (PAGA)

Hormone-associated differentially expressed genes (HA-DEGs)

Multiplexed error-robust fluorescence in situ hybridization (MERFISH) differential gene expression (DE)

Differentially expressed genes (DEGs)

Support vector machine (SVM)

Manifold Enhancement Latent Dimension (MELD)

Potential of Heat-diffusion for Affinity-based Trajectory Embedding (PHATE)

*Androgen receptor (AR)*

single-cell regulatory network inference (SCENIC)

**Reviewer #3 (Public review):**

We appreciate reviewer for the constructive comments to improve our manuscript.

Weaknesses:

We already know that Esr1 is important within GABAergic but not glutamatergic neurons for mating behavior. However, there is not enough data to support the claim that disrupting Esr1 in glutamatergic MPOA neurons "had no observable effect." The MPOA is involved in many behaviors and physiologies that were not investigated. More assays would be required to report "no observable effect."

The small number of cells included in the transcriptional studies is a general concern, as noted by the authors. This is a particular concern for conclusions related to the role of adolescence in glutamatergic MPOA neurons. The paper reports 24,627 neurons across all treatment groups, which include 3 time points, 2 sexes, and GDX conditions. It seems likely that not much was detected in the glutamatergic neurons because of insufficient power.

Esr1 knockout is initiated in adolescence, not restricted to adolescence. Do we know that the effects on mating behavior are due to what is happening in adolescence vs. the function of Esr1 in adults? Are the effects different if Esr1 is knocked out in mature adults? This comparison would be important to demonstrate that adolescence is a critical time window for Esr1 function.

We agree that 1. the relatively mild effects observed in Glutamatergic neurons may be partially due to the scale of the study, and 2. *Esr1* deletion is permanent once induced and it is challenging to distinguish adolescent and adult transcriptional dynamics using existing viral strategies.

We added discussion in the “limitation” section.

“4. While we have observed robust transcriptional progression in *Vgat^+^ Esr1^+^* neurons during adolescence, we observed more mild alternations in *VgluT2^+^* neurons. Although the scale of our study is comparable or exceeds prior scRNAseq studies in MPOA[22,29], future larger studies may have more sensitivity to detect adolescent transcriptional dynamics in *VgluT2^+^* neurons.”

“5. Although we demonstrated adolescent transcriptional changes were observed as early as *P35*, and either hormonal deprivation or *Esr1 KO* in prior to adolescence prevented the transcriptional progression (arrested transcriptional state even at adult), given the viral incubation time and permanent deletion of *Esr1* after viral injection, it is challenging to disambiguate the role of *Esr1* during adolescence and adult. Future studies injecting the virus at adult may provide additional insights on the similarity and difference between transcriptional changes during puberty and maintained transcriptional states at adult.”
